# Gold marker displacement due to needle insertion during HDR-brachytherapy for treatment of prostate cancer: A prospective cone beam computed tomography and kilovoltage on-board imaging (kV-OBI) study

**DOI:** 10.1186/1748-717X-7-24

**Published:** 2012-02-20

**Authors:** Markus KA Herrmann, Tereza Kertesz, Tammo Gsänger, Eugen Bloch, Gerhard Pollul, Mohamed Bouabdallaoui, Arne Strauss, Mareike Herrmann, Hans Christiansen, Hendrik A Wolff, Clemens F Hess, Andrea Hille

**Affiliations:** 1 Department of Radiotherapy, University of Goettingen, Goettingen, Germany; 2 Department of Urology, University of Goettingen, Goettingen, Germany

**Keywords:** Prostate cancer, HDR-brachytherapy, Gold markers, Conformal radiotherapy, Intensity modulated radiotherapy

## Abstract

**Purpose:**

To evaluate gold marker displacement due to needle insertion during HDR-brachytherapy for therapy of prostate cancer.

**Patients and methods:**

18 patients entered into this prospective evaluation. Three gold markers were implanted into the prostate during the first HDR-brachytherapy procedure after the irradiation was administered. Three days after marker implantation all patients had a CT-scan for planning purpose of the percutaneous irradiation. Marker localization was defined on the digitally-reconstructed-radiographs (DRR) for daily (VMAT technique) or weekly (IMRT) set-up error correction. Percutaneous therapy started one week after first HDR-brachytherapy. After the second HDR-brachytherapy, two weeks after first HDR-brachtherapy, a cone-beam CT-scan was done to evaluate marker displacement due to needle insertion. In case of marker displacement, the actual positions of the gold markers were adjusted on the DRR.

**Results:**

The value of the gold marker displacement due to the second HDR-brachytherapy was analyzed in all patients and for each gold marker by comparison of the marker positions in the prostate after soft tissue registration of the prostate of the CT-scans prior the first and second HDR-brachytherapy. The maximum deviation was 5 mm, 7 mm and 12 mm for the anterior-posterior, lateral and superior-inferior direction. At least one marker in each patient showed a significant displacement and therefore new marker positions were adjusted on the DRRs for the ongoing percutaneous therapy.

**Conclusions:**

Needle insertion in the prostate due to HDR-brachytherapy can lead to gold marker displacements. Therefore, it is necessary to verify the actual position of markers after the second HDR-brachytherapy. In case of significant deviations, a new DRR with the adjusted marker positions should be generated for precise positioning during the ongoing percutaneous irradiation.

## Introduction

Modern radiation therapy procedures increasingly utilize three-dimensional delivery techniques, which necessitate accurate patient positioning. An improved dose delivery by high precision technique, such as intensity modulated therapy (IMRT or VMAT) requires an even higher degree of accuracy in patient positioning. Therefore, minimization of set-up errors and localization uncertainties of the prostate within the patient have become critical components of highly conformal radiotherapy, especially in IMRT and VMAT. The use of gold markers implanted in the prostate and visualized daily using kV-OBI imaging has therefore been employed to quantify organ position variation and provide an accurate and efficient method for prostate localization [1-5].

High-dose-rate (HDR)-brachytherapy is a highly precise option in prostate cancer treatment to deliver high doses of radiation to the prostate as a boost to percutaneous irradiation, as monotherapy in the primary treatment setting or as salvage therapy [6-16]. In the combined treatment setting various fractionation schedules and implant timing schedules of the HDR-brachytherapy have been described [17-21]. If HDR-brachytherapy is used in the combined treatment, a further invasive approach for the implantation of the gold marker in the prostate, which can result in possible side effects such as bleeding and infection [22], should be avoided. Therefore, the implantation of the gold markers in to the prostate gland should be performed at the end of the first HDR-brachytherapy before starting the planning for percutaneous irradiation.

When percutaneous therapy is started before completion of the HDR-brachytherapy fractions, a gold marker displacement by the needle insertion into the prostate gland during HDR-brachytherapy should be taken into account and the gold marker localization should be controlled.

The purpose of the current prospective evaluation was to assess quantitatively gold marker displacement by needle insertion during HDR-brachytherapy in the treatment of prostate cancer. To our knowledge, this is the first prospective analysis of the effect of needle insertion due to HDR-brachytherapy on gold marker localization within the prostate in the percutaneous treatment of prostate cancer.

## Methods and materials

### Patients

18 sequential patients who underwent radiotherapy for prostate cancer were enrolled in this prospective evaluation. Inclusion criteria were defined as follows: prostate carcinoma stages T2b-T3a or stages T1-T2a with Gleason-score ≥7 and/or PSA ≥10 ng/ml. Patients who met these criteria and which were intended to receive combined percutaneous radiotherapy and HDR-brachytherapy were eligible for the study. Patient characteristics are shown in Table 1. The primary tumor was staged clinically, according to the TNM classification of 2003 for prostate cancer [23]. Staging examination included a physical examination of all patients. Furthermore, abdominal ultrasound or CT-scans of the abdomen, CT-scan of the pelvis, chest X-ray, and bone scan were done in patients with PSA-levels ≥20 ng/ml or stage T3. All Patients were instructed per protocol to empty the rectum by initiation of a special diet and medication against flatulence 2 weeks before the beginning of the combined treatment.

**Table 1:** Characteristics of patients entered this in study

Age	72,5 (57-77)
**Tumor stage**	

T1c	3 (17%)

T2a	2 (11%)

T2b	3 (17%)

T2c	8 (44%)

T3a	2 (11%)

**Nodal stage**	

N0	16 (89%)

N+	2 (11%)

**Gleason Score**	

≤ 6	9 (50%)

7	6 (33%)

≥ 8	3 (17%)

**PSA-Level initial**	

< 10	10 (56%)

10-20	1 (6%)

> 20	7 (39%)

**Previous treatment**	

no hormonal ablative therapy	9 (50%)

hormonal ablative therapy	9 (50%)

**Treatment volume**	

prostate only	12 (67%)

whole pelvis	6 (33%)

### Treatment

The first HDR-brachytherapy was the first treatment in all patients. Three gold marker were implanted transperineally into the prostate via a biopsy needle under ultrasound guidance at the end of the HDR-brachytherapy procedure. The dimensions of the cylinder-shaped striated soft-tissue gold markers were 1.2 mm in diameter and 3 mm in height with a volume of 3.4 mm^3 ^(Additec, Hannover, Germany).

HDR-brachytherapy was done with the Swift Oncentra Brachytherapy treatment planning software (Swift Oncentra prostate, Version 3.0, Nucletron Corporation B.V., Veenendaal, The Netherlands). Patients received two fractions with each 9 Gy in a two week interval. The implantation of the HDR needles as well as the gold marker was performed by a trained urologist using a 7.5 MHz TRUS probe (BK medical, Herlev, Denmark). Planning was based upon 3D ultrasound images, therefore axial cross sections were acquired in 1 mm steps and transferred to the planning software (Swift Oncentra Prostate, version 3.0, Nucletron Corporation B.V., Veendendal, The Netherlands). Contours of the prostate, urethra and rectum were delineated in all slices by the urologist and the radio-oncologist, whereas the PTV was defined by the radio-oncologist as the entire prostate gland without margins. The conformal treatment plan was created during the implantation by implying a predefined standard needle distribution, commonly used for HDR-brachytherapy in our department. The plan was generated according to the following requirements and constrains: the prescribed dose was specified to the PTV surface, the maximum dose to the urethra should not exceed 125%, and 2% of the rectum volume should not exceed 75% of the total dose.

Three days later, a CT-scan (Siemens Somatom Balance, Erlangen, Germany) was acquired using 2 mm slices for planning of the external beam irradiation. Patients were instructed to arrive for the planning CT-scan with a full bladder and an empty rectum as described before. Patient positioning on the treatment table for the external beam radiotherapy was depending on the indication for either a whole pelvic treatment or a prostate gland only treatment, derived from tumor stage, gleason-score, nodal status and PSA serum-levels. Therefore, for external beam radiotherapy of the prostate only a supine position was chosen, whereas the irradiation of the whole pelvis was done in a prone position with a belly-board. No special patient selection was performed for the patients analyzed in this study.

3D conformal computer-based planning was performed on the Eclipse/Aria treatment planning system (Eclipse/Aria external beam planning, Version 8.2.24, Varian medical systems, Palo Alto, USA). The prostate and proximal seminal vesicles were defined as the CTV. The PTV was defined as the CTV with an additional safety margin of 7 mm in each direction. Additionally, in patients scheduled for pelvic lymph node irradiation, the lymph nodes were defined and a safety margin of 10 mm in each direction was added for treatment planning. For all Patients, a dosage of 50 Gy, with 2 Gy per fraction, was prescribed. The irradiation technique included individual optimization with conformal treatment planning and the use of a multiple field technique with individual MLC (Multileaf Collimators) contours, or intensity modulated (IMRT or VMAT) technique. Dose was specified according to the ICRU 50 report [24]. After the treatment planning, one pair of orthogonal (anterior-posterior and lateral) DRRs was generated and the position of the gold markers could be defined. This image set constituted the reference image pair and was exported from the treatment planning system to the treatment station, together with the patient treatment plan.

One week after the first HDR-brachytherapy, percutaneous radiotherapy was initiated. For the verification of the patient positioning during the percutaneous treatment, verification images were taken daily (VMAT) or weekly (IMRT, 3-D conformal multiple field-technique) by the kilovoltage on-board imaging (kV-OBI) system (Varian medical systems, Palo Alto, USA) as a matter of routine in our department. Subsequently, these verification images were compared to the digitally reconstructed radiograph (DRR), generated during the planning process, by a radio-oncologist with special regard to the location of the markers was defined. Depending on the gold marker deviation between the verification images during treatment and the DRR, set-up corrections for the patient positioning were performed.

The second HDR-brachytherapy was performed as described above. Although the gold markers were already placed and localized prior to the insertion of the needles, there was no block-out of the areas with gold markers for needle insertion. After the second HDR-brachytherapy, a kilovoltage cone-beam CT-scan (CBCT) was performed. Subsequently, the CBCT-scan and the initial CT-scan were compared by soft tissue registration of the prostate. The positions of the markers within the prostate were documented on each scan and the displacement values were analyzed. Due to the changes of the prostate gland in shape and volume after the second HDR-brachytherapy we used the apex of the prostate gland as origin for the soft tissue registration, because the apex is, in comparison to other structures involved, less dependent on internal influences as changing bladder and rectal fillings. Accordingly, the registration process and the analysis were performed by the same radio-oncologist to get an optimal coverage of the two volumes. In case of marker displacement, the actual positions of the gold markers were transferred to the DRR for correction during the following irradiation fractions.

### Statistical analysis

Analysis was performed using the program STATISTICA 7.0 (Stat Soft, Palo Alto, USA). For evaluation of the statistical significance (values < 0.05) of differences, either the Wilcoxon-matched-pairs test or the Mann-Withney-U test was performed. For the quantification of the gold marker displacements, statistical data such as the mean, standard deviation (SD) and minimum and maximum displacement values were calculated. The gold marker displacement after the second HDR-brachytherapy (CBCT) as well as the daily or weekly gold marker deviations during irradiation (kV-OBI) have been analyzed and compared. For each patient, the mean gold marker deviation was calculated by analysis of the average deviations measured on each kV-OBI image performed during the external beam radiotherapy. We did not distinguish between random and systematic errors. The associated standard deviation was also calculated. Furthermore, the mean displacement of the entire group was computed by averaging over all patients.

## Results

18 patients were included into this prospective evaluation.

### Gold marker displacement analysis during external beam radiotherapy

A total of 214 kV-OBI images were acquired over the course of the study. For all patients, the maxima, minima, means and standard deviations of the magnitude of gold marker displacement were as follows: for anterior-posterior (AP) 19 mm, 0.0 mm, -1.6 ± 5 mm, for lateral (LR) 14 mm, 0.0 mm, 0.6 ± 4 mm, for superior-inferior (SI) directions 11 mm, 0.0 mm, 0.8 ± 5 mm.

The maxima, minima, means and standard deviations of the magnitude of gold marker displacement in patients receiving percutaneous radiotherapy for the prostate only (n = 154 kV-OBI images), were as follows: for anterior-posterior (AP) -13 mm, 0.0 mm, -2 ± 4 mm, for lateral (LR) 12 mm, 0.0 mm, 1 ± 4 mm, and for superior-inferior (SI) directions 8 mm, 0.0 mm, 1 ± 4 mm (Table 1).

The maxima, minima, means and standard deviations of the magnitude of gold marker displacement in patients receiving external beam radiotherapy of the pelvic nodes (n = 60 kV-OBI images) were as follows: for anterior-posterior (AP) 19 mm, 0.0 mm, -0.6 ± 5 mm, for lateral (LR) -14 mm, 0.0 mm, -0.6 ± 4 mm, and superior-inferior (SI) directions -8 mm, 0.0 mm, -1 ± 4 mm (Table 1).

### Gold marker displacement analysis after HDR-brachytherapy

A total of 54 gold markers were implanted. In every patient, a CBCT-scan was acquired after the second HDR-brachytherapy, prior to the following external beam irradiation fraction. During the first week of external beam radiotherapy, prior the second HDR-brachytherapy, the marker position showed no significant deviation or migration on the verification images in comparison to the DRRs of the planning CT-scans.

Following the second HDR-brachytherapy, the displacement of each gold marker within the prostate was analyzed after registration of the CBCT to the planning CT-scan. The maxima, minima, means and standard deviations of the magnitude of gold marker displacement were as follows: for anterior-posterior (AP) 5 mm, 0.0 mm, -0.4 ± 2 mm, for lateral (LR) -7 mm, 0.0 mm, 0.4 ± 2 mm, and for superior-inferior (SI) directions 12 mm, 0.0 mm, -3 ± 4 mm (Table 2). Our analysis of the magnitude of gold marker displacement between the two brachytherapy procedures showed significant differences in the superior-inferior (SI) direction compared as well to the anterior-posterior (AP) direction (*p *= 0.0007) as to the lateral (LR) direction (*p *= 0.00003). No statistical significant difference was found in the anterior-posterior direction compared to the lateral direction (*p *= 0.08).

**Table 2:** Error of gold marker displacement after HDR-brachytherapy and during irradiation for each patient

Patient	gold marker displacement after second HDR-brachytherapy			gold marker displacement during external beam radiotherapy		
	**Superior-inferior** **maximum, minimum, mean, SD (mm)**	**Anterior-posterior** **maximum, minimum, mean, SD (mm)**	**Left-right** **maximum, minimum, mean SD (mm)**	**Superior-inferior** **maximum, minimum, mean, SD (mm)**	**Anterior-posterior** **maximum,minimum, mean SD (mm)**	**Left-right** **maximum, minimum, mean SD (mm)**

**1**	**2, 0.0, 0.7 ± 1**	**0.0, 0.0,0.0 ± 0.0**	**2, 0.0, 2 ± 1**	**4, 0.0, 0.2 ± 3**	**-5, 0.0, 0.8 ± 4**	**-4, 2, -2 ± 3**

**2**	**0.0, 0.0, 0.0 ± 0.0**	**-2, 0.0, -0.7 ± 1**	**-2, 0.0, -0.7 ± 1**	**8, 0.0, 1 ± 7**	**-4, 0.0, -2 ± 2**	**-4, -1, -3 ± 2**

**3**	**2, 0.0, 0.7 ± 1**	**5, 4, 5 ± 0.6**	**-3, -1, -2 ± 1**	**-5, 0.0, -0.6 ± 2**	**-13, 0.0, -4 ± 3**	**8, -3, 2 ± 0.3**

**4**	**2, 0.0, 0.7 ± 1**	**-2, 0.0, -7 ± 1**	**0.0, 0.0, 0.0 ± 0.0**	**-6, -2, -4 ± 2**	**-6, 0.0, -3 ± 2**	**-7, 0.0, -3 ± 3**

**5**	**12, 6, 9 ± 3**	**-1, 0.0, -0.3 ± 0.6**	**3, -2, 1 ± 3**	**11, 0.0, 4 ± 5**	**-6, 0.0, -2 ± 3**	**-8, 0.0, -4 ± 4**

**6**	**2, 0.0, 1 ± 1**	**0.0, 0.0, 0.0 ± 0.0**	**-1, 0.0, -0.3 ± 0.6**	**3, 0.0, 1 ± 2**	**-7, 0.0, -4 ± 2**	**3, 0.0, 0.1 ± 1**

**7**	**6, 6, 6 ± 0.0**	**0.0, 0.0, 0.0 ± 0.0**	**-1, 0.0, -0.3 ± 0.6**	**-6, 0.0, -2 ± 3**	**6, 0.0, 0.0 ± 4**	**4, 0.0, 1 ± 2**

**8**	**12, 2, 8 ± 5**	**4, 3, 4 ± 0.6**	**-3, 0.0, -2 ± 2**	**11, -2, 5 ± 7**	**-6, 0.0, -2 ± 3**	**-8, 0.0, -3 ± 4**

**9**	**6, -2, 1 ± 4**	**-3, 0.0, -1 ± 2**	**2, 0.0, 0.7 ± 1**	**11, -1, -6 ± 4**	**12, 0.0, -15 ± 4**	**-10, 0.0, 2 ± 4**

**10**	**8, 2, 6 ± 3**	**-2, 0.0, -0.7 ± 1**	**-7, 0.0, -2 ± 4**	**-11, -5, -7 ± 3**	**-8, 0.0, -5 ± 4**	**-9, 0.0, -6 ± 4**

**11**	**2, 0.0, 0.7 ± 1**	**0.0, 0.0, 0.0 ± 0.0**	**0.0, 0.0, 0.0 ± 0.0**	**7, 0.0, 0.4 ± 4**	**-8, 0.0, -0.6 ± 4**	**-8, -2, -3 ± 3**

**12**	**2, 0.0, 0.7 ± 1**	**-3, 0.0, -1 ± 2**	**-4. 0.0, -2 ± 2**	**-5, 0.0, 0.8 ± 3**	**-5, 2, 0.2 ± 10**	**-3, 0.0, 0.0 ± 2**

**13**	**-2, -2, -2 ± 0.0**	**4, 0.0, 1 ± 2**	**-3, 0.0, 1 ± 2**	**-8, 0.0, -5 ± 3**	**-5, 0.0, -1 ± 2**	**-9, 0.0, -0.6 ± 4**

**14**	**8, 6, 7 ± 1**	**5, 4, 4 ± 0.6**	**-3, 1, -1 ± 2**	**-9, 1, 1 ± 4**	**19, -1, 3 ± 8**	**-14, -2, 0.7 ± 7**

**15**	**0.0, 0.0, 0.0 ± 0.0**	**-3, 0.0 -1 ± 2**	**4, 0.0, 2 ± 2**	**8, 0.0, 2 ± 3**	**-13, -1, -3 ± 5**	**12, 0.0, 3 ± 3**

**16**	**8, -4, 0.0 ± 7**	**-4, 2, -1 ± 3**	**-2, -1, -1 ± 0.6**	**-8, 0.0, -4 ± 4**	**-6, 1, -3 ± 3**	**9, 1, 4 ± 3**

**17**	**4, 3, 4 ± 0.6**	**0.0, 0.0, 0.0 ± 0.0**	**-1, 0.0, -0.3 ± 0.6**	**5, 1, 2 ± 2**	**10, 2, 34**	**3, -1, 0.3 ± 2**

**18**	**5, 0.0, 2 ± 3**	**0.0, 0.0, 0.0 ± 0.0**	**0.0, 0.0, 0.0 ± 0.0**	**11, 1, 5 ± 3**	**-13, 0.0, -0.6 ± 5**	**8, 1, 5 ± 2**

### Comparison of the gold marker localization changes after the second HDR-brachytherapy and during the following percutaneous irradiation

The magnitude of gold marker deviation in the SI direction was much more pronounced in the analysis after the second HDR-brachytherapy than during the following external beam radiotherapy period for both pelvic node and prostate only irradiation. However, this difference was not statistical significant. Analysis of the gold marker deviation values in the AP and LR direction showed contrary results. The deviation values for both directions were statistical significant lower in the analysis after the second HDR-brachytherapy compared to the deviation values in the analysis during the following external beam radiotherapy, for both pelvic node (*p *= 0.007) and (*p *= 0.02) and prostate only irradiation (*p *= 0.0002) and (*p *= 0.00004), respectively.

## Discussion

Modern percutaneous radiation therapy procedures increasingly utilize three-dimensional (3D) conformal or intensity-modulated (IMRT or VMAT) techniques, which necessitate a maximum degree of accuracy in patient positioning and tumour targeting. The use of radio-opaque gold markers implanted in the prostate gland and visualized daily by using kV-OBI imaging has therefore been employed. These markers provide the quantification of organ position variations and therefore allow for a reliable coverage of the target by increased accuracy in prostate localization [1-5]. However, when gold markers are used, at least two aspects have to be considered, on the one hand gold marker migration itself within the prostate gland and on the other hand gold marker migration by deviation of the whole prostate gland within the small bowel because of external influences, e.g. filling of the bladder or the rectum. Therefore, gold marker migration has been studied by several investigators to quantify and characterize the movements of the markers within the prostate gland. It has been established that there is a migration of the markers within the prostate gland, but this deviations were not shown to be significant [25,26]. Furthermore, gold marker displacement due to prostate movement during external beam radiotherapy has been analyzed in many studies as well [1-5].

The regular magnitude of displacement of the gold marker during external beam radiotherapy showed comparable values in the superior-inferior direction and significant higher values in the anterior-posterior and the lateral direction. The analysis of the marker displacement values during percutaneous radiotherapy shows the corresponding displacements values as described before, caused by inter- and intrafraction motion of the whole prostate gland itself due to multifaceted external and internal influencing factors [1-5]. The aspect of a possible marker displacement or movement due to needle insertion during a HDR-brachytherapy is a further important source of interference in the use of gold markers for prostate localization. The impact of the needle insertion on the position of gold markers within the prostate gland and its relevance for the accuracy of patient positioning during the following percutaneous treatment has not been considered or analyzed until now.

For the evaluation of the gold marker deviations the CT-scan after the first HDR-brachytherapy and the CBCT-scan after the second HDR-brachytherapy were registered and analyzed by the same radio-oncologist during a replanning procedure. Soft tissue matching of the prostate gland was performed on every patient for the analysis of the marker deviations. Due to the changes of the prostate gland in shape and volume after the second HDR-brachytherapy in all but 2 cases (Table 3), the registration process was done according to predefined specifications in our department. We defined the apex of the prostate gland as the origin for the soft tissue registration of the prostate, to achieve reliable, accurate and comparable results. Among all involved structures in the region of the small bowel, the apex appears to be less dependent on internal influences as different bladder and rectal fillings. In view of the general difficulties in prostate localization in CT-scans during a normal planning process, our predefined specifications for the soft tissue matching of the prostate gland proved to be reliable, comparable and safe in daily practice.

**Table 3:** Volume changes of the prostate gland for each patient between CT-scan after first HDR and CBCT-scan after second HDR-brachytherapy

Patient	First HDR-brachytherapy		Second HDR-brachytherapy	
	**Number of needles used**	**Volume of the prostate gland (ml)**	**Number of needles used**	**Volume of the prostate gland (ml)**

**1**	17	45	18	52

**2**	17	45	18	45

**3**	14	20	17	27

**4**	14	18	15	21

**5**	15	24	16	39

**6**	15	31	18	37

**7**	16	37	18	56

**8**	16	22	17	27

**9**	18	66	18	89

**10**	16	33	17	38

**11**	18	51	17	42

**12**	18	22	17	24

**13**	18	40	17	54

**14**	16	34	17	26

**15**	17	57	18	69

**16**	18	23	17	38

**17**	16	22	18	28

**18**	14	28	17	45

Our data showed gold marker deviations in all directions for each patient, as well in the analysis of the marker localizations after the second HDR-brachytherapy as in the analysis during the following external beam radiotherapy. The analysis of the gold marker displacements after the second HDR-brachytherapy in each patient before continuing external beam irradiation showed statistically significant deviation values in the superior-inferior direction compared to both the anterior-posterior and lateral direction. Therefore, needle insertion through the perineum into the prostate gland consequentially results in a deviation of the markers which follows the direction of the needles inserted during HDR-brachytherapy.

Possible reasons for the displacement of the gold markers caused by insertion of needles during HDR-brachytherapy are localized bleedings into the tissue of the prostate gland, or a dislocation of prostate tissue inside the gland due to needle insertion. Furthermore, a direct contact between the gold marker and the inserted needle should be considered as possible explanation.

Our data show, that gold marker displacement eithin the prostate gland can be caused by HDR-brachytherapy. This displacement could possibly result in an insufficient percutaneous treatment with incomplete covering of the target because of an incorrect marker based image guidance, especially the prostate within small safety margin (7 mm), when actual marker positions were not considered.

In some patients the displacement was more pronounced than in other patients (Table 3). This could be due to different prostate volumes and needle insertions. The prostate volume during the first and second HDR-brachytherapy was in minimum 18 and 21 cm^3^, in maximum 66 and 89 cm^3 ^and in mean 32 and 42 cm^3 ^respectively. The quantity of needles used for the HDR-brachytherapy was in minimum 14 and 15, in maximum 18 and 17 and in mean 16 and 17, respectively. We both observed an increase (14 patients), a decrease (2 patients) and no change (2 patients) in prostate volumes after HDR-brachytherapy including application of the gold markers, as it has previously been published by our department [27]. In the analyzed 18 patients no correlation was seen between the volume change of prostate and magnitude of seed gold marker displacement. Though we did not find a difference in the displacement values between small and large prostate volumes this could be more pointed out in a larger patient population.

Our study included only a small number of patients, but a significant gold marker displacement was observed in the superior-inferior direction compared to the anterior-posterior and lateral directions after the second HDR-brachtherapy in some patients. In conclusion, the control and subsequent adjustment of the actual marker localization on the DRR after HDR-brachytherapy is necessary for precise correction during the following percutaneuos treatment, whereas it is also possible to initiate a complete new planning process using new planning CT-scans. Another option is the implementation of a different treatment schedule. The completion of HDR-brachytherapy implants prior to the planning of the external beam radiotherapy avoids additional gold marker displacements due to manipulations of the prostate gland during external beam irradiation, as we do now as standard approach in our department.

## Competing interests

No actual or potential competing exist.

## Authors' contributions

MKAH, planning of HDR therapy and performance of HDR therapy, drafting the manuscript, corresponding author. TK, planning percutaneous treatment, drafting the manuscript. TG, medical physicists, planning HDR treatment and percutaneous treatment. EB, planning of HDR therapy and performance of HDR therapy. GP, medical physicists, planning HDR treatment and percutaneous treatment. MB, medical physicist, planning HDR treatment and percutaneous treatment. AS, Urologist, performed the HDR Needle insertion. MH, Urologist, performed the HDR Needle insertion. HC, responsible physician during percutaneous therapy. HAW, planning percutaneous treatment, responsible physician during percutaneous therapy. CFH, responsible physician during percutaneous therapy. AH, planning of HDR therapy, drafting the manuscript. All authors read and approved the final Manuscript.
